# Relationship Between Perceived Physical and Mental Fatigability and Physical Performance in Very Old Adults

**DOI:** 10.1093/geroni/igaa057.576

**Published:** 2020-12-16

**Authors:** Yixin Hu, Zhuangzhuang Zhang, Woei-Nan Bair, Anying Bai, Li Fan

**Affiliations:** 1 Chinese PLA General Hospital, Beijing, China; 2 Peking University Health Science Center, Beijing, China; 3 University of the Sciences in Philadelphia, Philadelphia, United States; 4 Peking University, Beijing, China; 5 Chinese PLA hospital, Beijing, China

## Abstract

To investigate the relationship between perceived physical and mental fatigability and physical performance in community-dwelling very old adults (≥80 years). We examined the association in one retired community in Beijing including 404 very old adults. Pittsburgh Fatigability Scale (PFS), Chinese version, was used to assess perceived fatigability in physical domain (PFS-P) and mental domain (PFS-M). High fatigability is defined as PFS-P ≥ 15, and PFS-M ≥ 13. Physical performance measures include grip strength, usual gait speed, chair stand and Short Physical Performance Battery (SPPB) test. Women have higher PFS scores (both PFS-P & PFS-M) and higher prevalence of high fatigability than men. After adjusting for sex, usual gait speed and SPPB scores were significantly associated with PFS-P & PFS-M, while grip strength and chair stand performance were significantly associated with PFS-P only. After multivariable adjustment, usual gait speed (B=-3.745, P=0.021) and chair stand performance (B=0.335, P=0.005) were significantly associated with PFS-P, while usual gait speed (B=-2.656, P=0.006) and SPPB scores (B=-0.214, P=0.029) were significantly associated with PFS-M. Perceived physical and mental fatigability is highly prevalent in very older adults and they differ by sex. The significant associations between PFS scores and performance measures suggest that PFS is of potential clinical importance, especially when testing performance measures are not feasible. Utilization of PFS score can assist in identifying target populations who are at risk of reduced physical functions, such as older with depression, older women. Interventions to improve usual gait speed are likely to reduce both perceived physical and mental fatigability.

